# MicroRNA-34a Modulates c-Myc Transcriptional Complexes to Suppress Malignancy in Human Prostate Cancer Cells

**DOI:** 10.1371/journal.pone.0029722

**Published:** 2012-01-03

**Authors:** Soichiro Yamamura, Sharanjot Saini, Shahana Majid, Hiroshi Hirata, Koji Ueno, Guoren Deng, Rajvir Dahiya

**Affiliations:** Department of Urology, San Francisco Veterans Affairs Medical Center and University of California San Francisco, San Francisco, California, United States of America; University of Barcelona, Spain

## Abstract

MicroRNA-34a (miR-34a), a potent mediator of tumor suppressor p53, has been reported to function as a tumor suppressor and miR-34a was found to be downregulated in prostate cancer tissues. We studied the functional effects of miR-34a on c-Myc transcriptional complexes in PC-3 prostate cancer cells. Transfection of miR-34a into PC-3 cells strongly inhibited *in vitro* cell proliferation, cell invasion and promoted apoptosis. Transfection of miR-34a into PC-3 cells also significantly inhibited *in vivo* xenograft tumor growth in nude mice. miR-34a downregulated expression of c-Myc oncogene by targeting its 3′ UTR as shown by luciferase reporter assays. miR-34a was found to repress RhoA, a regulator of cell migration and invasion, by suppressing c-Myc–Skp2–Miz1 transcriptional complex that activates RhoA. Overexpression of c-Myc reversed miR-34a suppression of RhoA expression, suggesting that miR-34a inhibits invasion by suppressing RhoA through c-Myc. miR-34a was also found to repress c-Myc-pTEFB transcription elongation complex, indicating one of the mechanisms by which miR-34a has profound effects on cellular function. This is the first report to document that miR-34a suppresses assembly and function of the c-Myc–Skp2–Miz1 complex that activates RhoA and the c-Myc-pTEFB complex that elongates transcription of various genes, suggesting a novel role of miR-34a in the regulation of transcription by c-Myc complex.

## Introduction

MicroRNAs (miRNAs) are highly conserved, single stranded, non-coding RNAs of approximately 22 nucleotides that regulate gene expression by posttranscriptional silencing of specific target mRNAs, by repressing translation or cleaving RNA transcripts [1]. miRNAs regulate diverse cellular processes such as cell-cycle progression, proliferation, apoptosis and development. miRNAs have been shown to function as oncogenes or tumor suppressor genes [2].

The p53 tumor suppressor is deleted or mutated in more than 50% of human tumors and is a key molecule which suppresses malignancies [3]. p53 has been found to target the miR-34 family [4], [5], [6] and the ectopic expression of miR-34 genes has drastic effects on cell proliferation and survival. Ectopic miR-34a causes cell-cycle arrest in the G1 phase [6], [7] and apoptosis [7], [8]. As p53 has been found to target miR-34a and since, cell-cycle arrest and apoptosis are also end points of p53 activation, the miR-34a gene may be a mediator of p53 function.

The proto-oncogene c-Myc regulates cell proliferation and transformation both transcriptionally and non-transcriptionally and is frequently deregulated in human cancers [9], [10]. c-Myc is a basic helix–loop–helix and leucine zipper transcription factor which binds to Enhancer Box elements (E-boxes) and activates the transcription of genes which stimulate cell cycle progression and cell growth. c-Myc suppresses the transcription of genes which arrest the cell cycle, through Miz1, the c-Myc associated protein. c-Myc also has a function to recruite histone acetyltransferases (HATs). c-Myc non-transcriptionally interacts with components of the replication machinery to positively regulate DNA synthesis, leading to genomic instabilities.

c-Myc was reported to activate MiR-17-92, a polycistronic microRNA cluster consisting of miR-17, 18a, 19a, 20a, 19b and 92a [11], [12]. miR-19 was found to be the principal oncogenic component of this cluster, targeting the tumour suppressor PTEN [13]. miR-34c has been shown to negatively regulate c-Myc in response to DNA damage and to inhibit c-Myc-induced DNA synthesis [14]. During oncogene-induced senescence, miR-34a was also found to target c-Myc [15].

Rho GTPases are small G proteins that regulate various cellular processes, including cytoskeletal dynamics, migration, vesicle trafficking, cell proliferation, apoptosis, and transcription [16], [17], [18]. Rho GTPases, their regulators, and their effectors have been suggested to control tumor formation and progression. RhoA has been shown to control cancer metastasis and progression [19], [20], [21]. Recently, c-Myc complex was found to activate the RhoA gene [22].

The positive transcription elongation factor b (P-TEFb) regulates the promoter-proximal pause release of the elongation phase of transcription by Pol II [23] and integrates mRNA synthesis with histone modification, pre-mRNA processing and mRNA export [24]. P-TEFb is composed of cyclin (CycT1, CycT2a, CycT2b or CycK) and cyclin-dependent kinase 9 (Cdk9) [23]. c-Myc interacts with cyclin T1 (CycT1), the regulatory component of P-TEFb, and controls the elongation phase of transcription by Pol II [25], [26], [27].

The c-Met pathway is dysregulated in most human malignancies and regulates tumor formation and progression [28], [29]. c-Met interacts with hepatocyte growth factor/scatter factor and has been implicated in tumor invasion and migration including PC-3 cells [30], [31], [32], [33], [34], [35].

We report here that miR-34a was downregulated in prostate cancer tissues. miR-34a inhibited cell proliferation *in vitro*, *in vivo* tumor growth and promoted apoptosis in prostate cancer cells. We also found miR-34a targets c-Met and inhibits PC-3 cell invasion. We investigated the effects of miR-34a on the two c-Myc transcriptional complexes. By targeting c-Myc, miR-34a reduced the c-Myc-Miz-Skp2 complex which induces RhoA transcription and inhibited cell invasion, showing that miR-34a indirectly regulates RhoA. miR-34a also suppressed the c-Myc-P-TEFb complex that plays a key role in controlling the elongation phase of transcription by RNA polymerase II (Pol II), indicating one of the mechanisms by which miR-34a has dramatic effects on cellular function. Our results demonstrate that in prostate cancer PC-3 cells miR-34a suppresses assembly and function of the c-Myc complex that activates or elongates transcription, revealing a novel role of miR-34a in the regulation of transcription by c-Myc.

## Results

### miR-34a expression is downregulated in prostate cancer

**Figure 1:**
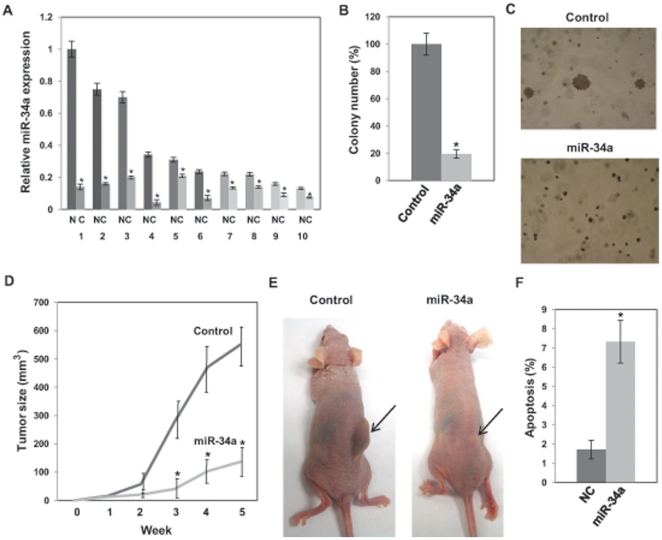
miR-34a inhibits cell growth. (A) Relative miR-34a expression in laser capture microdissected (LCM) prostate cancer tissues (C) and matched adjacent normal regions (N). (B) miR-34a overexpression suppresses soft agar colony formation of a stably transfected miR-34a PC-3 cell line. After 14 days incubation, colonies with over 50 cells were counted. The values are normalized to those of control. (C) Representative images of the colonies. (D) miR-34a inhibits *in vivo* tumor growth. Time course of tumor growth in nude mice after subcutaneous injection of a stably transfected miR-34a PC-3 cell line or control cell line. *, P<0.05 compared with control. (E) Representative images of tumors in nude mice at 5 weeks after subcutaneous injection of a stably transfected miR-34a PC-3 cell line or control cell line. (F) miR-34a induces apoptosis in PC-3 cells. PC-3 cells were transfected with pre-miR negative control (NC) or pre-miR-34a for 3 days. PC-3 cells were stained with AnnexinV-FITC/7-AAD and apoptosis was analyzed by flow cytometry. The data shows the percentage of early apoptotic and apoptotic cells out of the total cell population of PC-3 cells. *, P<0.05 compared with control.

We examined the expression levels of miR-34a in laser capture microdissected (LCM) prostate cancer tissues (n = 10) and matched adjacent normal regions by real-time PCR. All cancer tissues showed lower miR-34a levels compared with matched adjacent normal regions, demonstrating that miR-34a is downregulated in cancer (Fig. 1A). Histological data shows that these tissues are adenocarcinoma with Gleason scores of 6 or 7. Gleason patterns and other clinical data are shown in Table S1.

We also assayed miR-34a expression levels in malignant (PC-3, LNCaP and DU145 cells) and non-malignant prostate RWPE-1 cells. Real-time RT-PCR revealed that the expression level of miR-34a was markedly lower in PC-3 cells compared with non-malignant epithelial prostate cell RWPE-1 cells (data not shown). This result was consistent with the previously reported data using PrEC normal human prostate epithelial cells [36]. We also compared the expression levels of miR-34a in malignant prostate cells and the laser capture microdissected (LCM) prostate normal tissues (n = 10). Real-time RT-PCR showed that the expression level of miR-34a was lower in malignant prostate cells compared with the average of the expression level of miR-34a in the normal tissues (data not shown). These results were also consistent with the previously reported data using normal human tissues [37].

### miR-34a inhibits cell proliferation of PC-3 cells

To study the effect of miR-34a on the growth of prostate cancer cells, we transiently transfected several cell lines with pre-miR negative control (NC) or pre-miR-34a. The transient transfection of pre-miR-34a increased miR-34a levels in prostate cancer cells (Fig. S1A). MTS assay showed that miR-34a inhibited PC-3 cell proliferation by about 40% on day 4 but had no significant effect on LNCaP and DU145 cells (Fig. S2A). A recent report indicates that PC-3 cells have characteristics of prostatic small cell carcinoma and not of adenocarcinoma [38], however we used PC-3 cells for further study because the proliferation suppression effect of miR-34a was significant, which was consistent with the previously reported data [36]. Transient transfection of pre-miR-34a also caused significant morphological changes in PC-3 cells (Fig. S2B).

We employed a lentiviral system to express miR-34a. The HIV lentiviral system, expressing miR-34a or vector control, was used to infect PC-3 cells and the infected cells were selected with puromycin. The stable transfection of pre-miR-34a increased miR-34a levels in PC-3 cells (Fig. S1B). We performed soft agar colony formation assay using the infected PC-3 cells expressing miR-34a or control. miR-34a reduced colony numbers to about 20% of that of control, showing that miR-34a significantly inhibited the colony forming ability of PC-3 cells (Figs. 1B and C).

To examine the effects of the ectopic expression of miR-34a on *in vivo* tumor growth, we subcutaneously injected the stable miR-34a or the control cell line into nude mice. Tumor volumes were measured every 7 days for 5 weeks following the injection. Xenograft tumors from PC-3 cells overexpressing miR-34a were smaller than xenograft tumors from the control cells. At week 5, tumor sizes of control xenografts were about 5 times larger than those of miR-34a xenografts, indicating that the ectopic expression of miR-34a significantly suppressed tumor growth in vivo (Figs. 1D and E).

Since ectopic expression of miR-34a suppressed PC-3 cell proliferation, we next studied the effects of miR-34a on apoptosis in PC-3 cells using flow cytometry. We found that ectopic expression of miR-34a increased PC-3 cell apoptosis to about4 times of that controls (Fig.1F and Fig. S3), demonstrating that miR-34a has apoptotic activity in PC-3 cells.

### miR-34a inhibits cell invasion and migration

**Figure 2:**
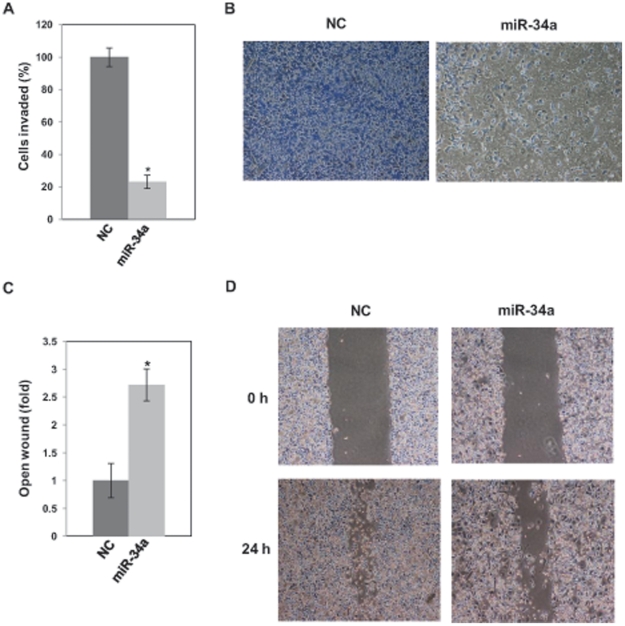
miR-34a inhibits invasion and migration of PC-3 cells. (A) miR-34a inhibits invasion of PC-3 cells. PC-3 cells were transiently transfected with pre-miR negative control (NC) or pre–miR-34a for 48 h. The cells were harvested and subjected to transwell invasion assay. The values are normalized to those of NC. *, P<0.05 compared with control. (B) Representative images of the invaded cells. (C) miR-34a inhibits wound-healing of PC-3 cells. PC-3 cells were transiently transfected with pre-miR negative control (NC) or pre–miR-34a for 48 h. The cells were harvested and subjected to wound-healing assay. The width of the remaining open wound calculated in relation to separation at time 0 h. *, P<0.05 compared with control. (D) Representative images of the wound healing assay.

We performed a transwell invasion assay using Matrigel to investigate the effect of miR-34a on the invasive ability of PC-3 cells. We transiently transfected PC-3 cells with pre-miR negative control (NC) or pre-miR-34a and subjected the transfected cells to transwell invasion assay. The results clearly revealed that miR-34 reduced invasion of PC-3 cells to 20% of that of controls (Figs. 2A and B). Wound-healing assay also showed that miR-34a markedly reduced the migration of PC-3 cells (Figs. 2C and D).

### miR-34a targets c-Myc and c-Met

**Figure 3:**
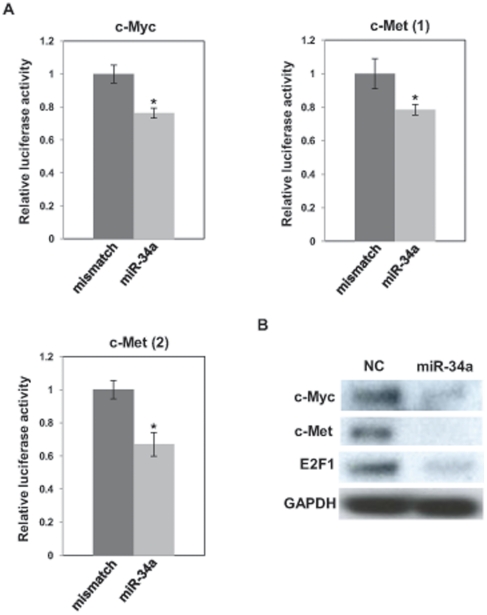
miR-34a targets oncogenes in PC-3 cells. (A) PC-3 cells were transiently transfected with pre-miR negative control (NC) or pre–miR-34a or pre–miR-con for 8 h, followed by transient transfection with control reporter plasmids or 3′UTR plasmids for 48 h. 3′UTR reporter activity was measured by luciferase assay and normalized to activity of Renilla luciferase. *, P<0.05 compared with control. (B) PC-3 cells were transiently transfected with pre-miR negative control (NC) or pre–miR-34a or pre–miR-con for 72 h. Protein expression level was analyzed by Western blot.

Oncogenes, c-Met and c-Myc, have predicted binding sites for miR-34a in their 3′-UTRs (Fig. S4). c-Met has 2 predicted binding sites for miR-34a in its 3′-UTRs (Fig. S3). We cloned the putative miR-34a targets in the 3′UTRs into a luciferase construct. Luciferase reporter assays with miR-34a-expressing PC-3 cells showed that miR-34a repressed luciferase activity. Mutation of the putative miR-34a target sites in these UTRs decreased the response to miR-34a. These results indicate that miR-34a binds to the 3′-UTRs of c-Myc and c-Met (Fig. 3A).

We examined the effects of miR-34a transfection on the protein levels of these genes. Transfection of miR-34a reduced the protein levels of c-Met and c-Myc, protein in PC-3 cells (Fig. 3B). These results confirm that miR-34a directly targets c-Met and c-Myc via binding to their 3′UTRs in PC-3 cells. miR-34a also reduced levels of transcription factor E2F1 which regulates the cell cycle, DNA replication and cell proliferation (Fig. 3B).

### miR-34a inhibits RhoA and suppresses assembly of c-Myc transcriptional complex

**Figure 4:**
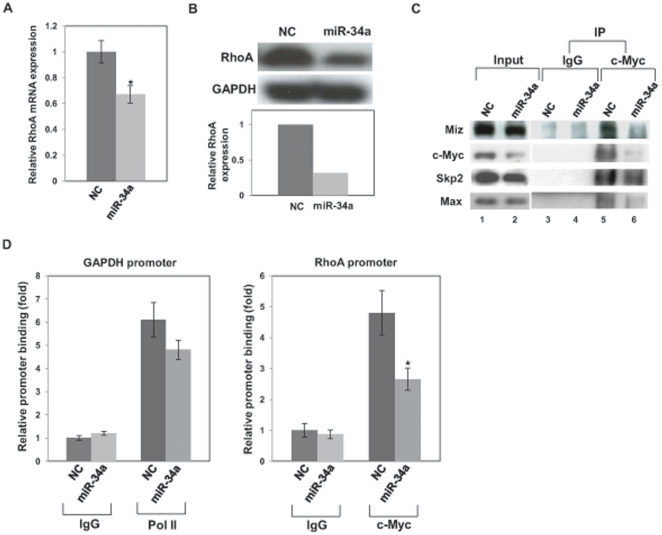
miR-34a suppresses the c-Myc transcriptional complex of RhoA. PC-3 cells were transiently transfected with pre-miR negative control (NC) or pre-miR-34a for 72 h. (A) RhoA mRNA level was analyzed by real-time PCR. (B) RhoA protein expression was analyzed by Western blot. (C) c-Myc pulls down endogenous Miz1, Skp2 and Max in PC-3 cells. PC-3 cells were transiently transfected with pre-miR negative control (NC) or pre–miR-34a for 72 h and total cell lysates were immunoprecipitated with c-Myc antibody or IgG (control), followed by Western blot analysis. (D) miR-34a decreases the recruitment of c-Myc to the RhoA promoter. PC-3 cells were transiently transfected with pre-miR negative control (NC) or pre–miR-34a for 72 h and ChIP assays were performed. *, P<0.05 compared with control.

The c-Myc–Skp2–Miz1 transcriptional complex has been found to activate RhoA gene and to be critical for cell invasion and cancer metastasis [22]. Since we found that c-Myc is a target of miR-34a, thus downregulating the expression of c-Myc, we examined whether the downregulation of c-Myc by miR-34a results in reduction of RhoA expression by suppressing assembly of the c-Myc–Skp2–Miz1 complex. Real-time PCR showed miR-34a decreased RhoA mRNA level (Fig. 4A) and Western blot revealed that miR-34a reduced RhoA protein expression (Fig. 4B).

We performed immunoprecipitation (IP) to study assembly of the c-Myc–Skp2–Miz1 transcriptional complex in miR-34a transfected cells. IP (Fig. 4C) revealed that anti-c-Myc immunoprecipitates contained Miz1, Skp2 and Max (lane 5) but not anti-IgG (control) immunoprecipitates (lane 3), indicating that the c-Myc–Skp2–Miz1 complex was formed in PC-3 cells. IP also showed that miR-34a decreased the levels of these components in the c-Myc immunoprecipitates [lanes 4 (control) and 6], indicating that miR-34a suppressed assembly of the c-Myc–Skp2–Miz1 transcriptional complex in PC-3 cells. miR-34a did not significantly reduce Skp2 in the immunoprecipitates compared with the other components.

We performed chromatin immunoprecipitation (ChIP) assays to examine whether miR-34a reduces binding of c-Myc to the RhoA promoter region containing E-boxes 5 and 6, which is a primary binding site of c-Myc [22]. The ChIP assay showed that endogenous c-Myc binds to E-boxes 5 and 6 and control experiments demonstrated that RNA polymerase II binding to the glyceraldehyde-3-phosphate dehydrogenase (GAPDH) promoter was not altered (Fig. 4D). These results suggested that miR-34a reduced the recruitment of the c-Myc–Skp2–Miz1 complex to the RhoA promoter and reduces RhoA expression.

### Overexpression of c-Myc reverses inhibition of invasion

**Figure 5:**
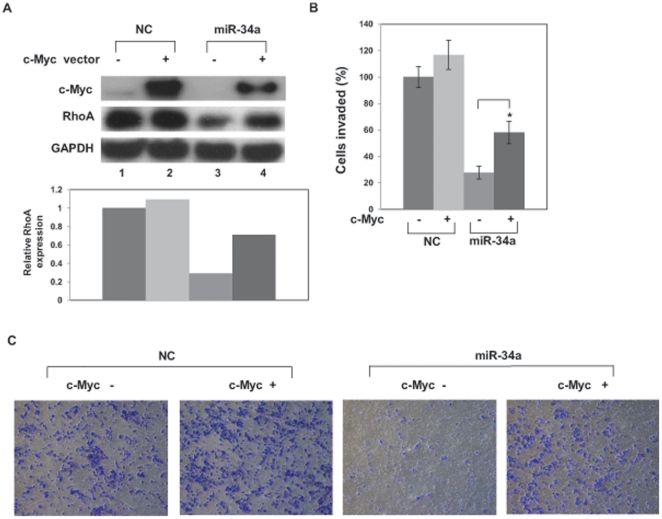
c-Myc overexpression partially rescues RhoA suppression by miR-34a. (A) PC-3 cells were transiently transfected with pCMV6-ENTRY only or pCMV6-ENTRY-c-Myc for 8 h followed by transient transfection with pre-miR negative control (NC) or pre–miR-34a for 72 h. Protein level was analyzed by Western blot. (B) c-Myc expression partially rescues inhibition of invasion induced by miR-34a. PC3 cells were transiently transfected with pCMV6-ENTRY only (control) or pCMV6-ENTRY-c-Myc for 8 h followed by transfection with pre-miR negative control (NC) or pre–miR-34a for 48 h. The cells were harvested and subjected to transwell invasion assay. *, P<0.05 compared with control. (C) Representative images of invaded cells.

To determine whether inhibition of invasion by miR-34a could be reversed via restoration of c-Myc, we transfected PC-3 cells with a c-Myc expression plasmid together with pre-miR-34a. c-Myc overexpression partially rescued RhoA expression (Fig. 5A, compare lane 4 with 3) and miR-34-induced suppression of invasion (Fig. 5B), suggesting that miR-34a inhibits invasion, at least partially, via RhoA reduction by targeting c-Myc.

### miR-34a suppresses assembly of c-Myc-P-TEFb complex

**Figure 6:**
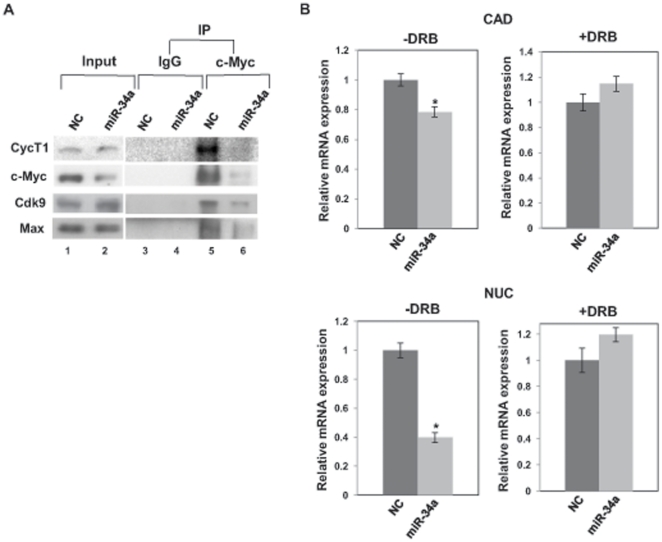
miR-34a suppresses the c-Myc-P-TEFb complex. (A) miR-34a suppresses the formation of the endogenous c-Myc-P-TEFb complex. PC-3 cells were transiently transfected with pre-miR negative control (NC) or pre–miR-34a for 72 h and total cell lysates were immunoprecipitated with c-Myc antibody or IgG (control), followed by Western blot analysis. (B) miR-34a suppresses CAD and NUC mRNA expression and DRB revereses the suppression. PC-3 cells were treated with 20 µM of DRB for 12 h and were transiently transfected with pre-miR negative control (NC) or pre–miR-34a for 48 h. CAD and NUC mRNA level was analyzed by real-time PCR.

The positive transcription elongation factor b (P-TEFb) plays a key role in controlling the elongation phase of transcription by RNA polymerase II (Pol II) [23]. It is a cyclin-dependent kinase comprised of Cdk9 andcyclin. c-Myc has been shown to interact with P-TEFb through CycT1 and regulate transcription elongation [25], [26], [27]. Since miR-34a targets c-Myc, we performed immunoprecipitation (IP) to study assembly of the c-Myc-pTEFB transcription elongation complex in mir-34a-transfected PC-3 cells. IP (Fig. 6A) revealed that anti-c-Myc antibody pulled down CycT1, Cdk9 and Max (lane 5) but IgG (control) did not pull down these proteins (lane 3), indicating that c-Myc interacted with P-TEFb. IP also showed that miR-34a decreased the amount of p-TEFb in the c-Myc immunoprecipitates in PC-3 cells [lanes 4 (control) and 6], indicating that miR-34a suppressed assembly of the c-Myc–P-TEFb complex in PC-3 cells.

c-Myc was reported to activate transcription of CAD, carbamoyl-phosphate synthase/aspartate transcarbamoylase/dihydroorotase, and NUC by stimulating promoter clearance and elongation, via recruitment of P-TEFb [25], [39], [40]. We studied whether miR-34a affected the expression level of CAD and NUC using DRB (5.6-di-chloro-1-b-D-ribofuranosyl-bensimidazole) which specifically suppresses expression of c-Myc-responsive CAD and NUC by inhibiting P-TEFb [40]. We found that miR-34a decreased CAD and NUC mRNA expression in PC-3 cells while DRB restored the expression (Fig. 6B). These results indicate that miR-34a repressed these genes by suppressing the c-Myc-pTEFb complex.

### Overexpression of c-Met reverses inhibition of invasion

**Figure 7:**
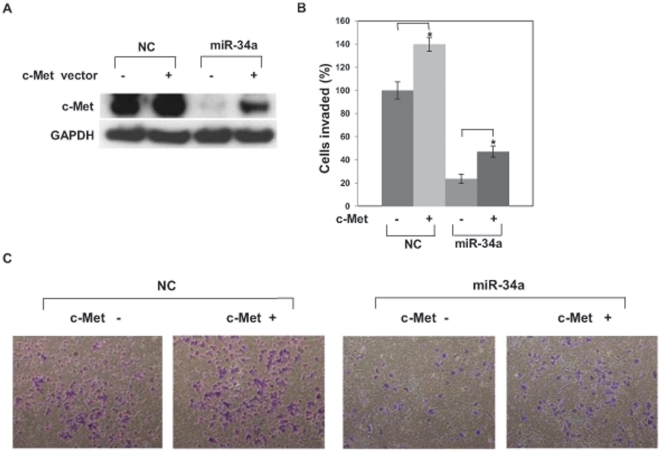
c-Met overexpression partially rescues suppression of cell invasion by miR-34a. (A) PC-3 cells were transiently transfected with pCMV6-ENTRY only or pCMV6-ENTRY-c-Met for 8 h followed by transient transfection with pre-miR negative control (NC) or pre–miR-34a for 72 h. Protein level was analyzed by Western blot. (B) c-Met expression partially rescues inhibition of invasion induced by miR-34a. PC3 cells were transiently transfected with pCMV6-ENTRY only (control) or pCMV6-ENTRY-c-Met for 8 h followed by transfection with pre-miR negative control (NC) or pre–miR-34a for 48 h. The cells were harvested and subjected to transwell invasion assay. *, P<0.05 compared with control. (C) Representative images of invaded cells.

It has been suggested that miR-34a inhibits cell invasion in a c-Met-dependent manner [41], [42]. Therefore, we studied effects of miR-34a on c-Met and invasion since miR-34a targets c-Met (Fig. 1). PC-3 cells were transfected with a c-Met expression plasmid together with pre-miR-34a. c-Met expression levels were examined by Western blot which indicated that c-Met expression was increased in the c-Met transfected cells (Fig. 7A). c-Met promoted invasion in control experiments. c-Met reversed miR-34-induced suppression of invasion, indicating that miR-34a inhibits invasion, at least partially, by targeting c-Met (Figs. 7B and C).

## Discussion

In this study we demonstrated for the first time the functional effects of miR-34a on c-Myc transcriptional complexes in PC-3 prostate cancer cells. We found that miR-34a expression is downregulated prostate cancer tissue and that miR-34a re-expression strongly inhibits cell proliferation, *in vivo* xenograft growth and cell invasion. We showed that miR-34a inhibited c-Myc by binding to its 3′UTR in PC-3 cells and suppressed assembly of the c-Myc transcriptional complexes. Mutations of c-Myc are found in various cancers and its deregulated expression causes the uncontrolled expression of many genes some of which regulate cell proliferation and results in tumor development [43], [44]. Although c-Myc alone is not able to transform cells and requires a co-operating oncogene such as ras oncogene [45], c-Myc is an important factor in tumor development. Thus, inhibition of c-Myc is thought to be a significant function of miR-34a. We also showed that miR-34a inhibited PC-3 cell invasion by targeting c-Met.

Rho GTPases, a family of small G proteins, regulate intracellular actin dynamics including cell polarity, migration, vesicle trafficking etc. These molecules also regulate cell proliferation, apoptosis, gene expression [17]. RhoA, a member of the Rho family of GTPases, is implicated in transformation and metastasis. RhoA has been shown to be an important factor associated with various human cancers [46].

The c-Myc–Skp2–Miz1 transcriptional complex has been previously shown to activate RhoA and to be essential for cell invasion and cancer metastasis [22]. We found that c-Myc is a target of miR-34a in PC-3 cells, and we studied the effect of miR-34a on RhoA activation via the c-Myc–Skp2–Miz1 transcriptional complex. miR-34a reduced RhoA expression and overexpression of c-Myc reversed the reduction of RhoA and inhibition of cell invasion. ChIP assay revealed miR-34a suppresses the recruitment of c-Myc to the RhoA promoter. We demonstrated that miR-34a suppressed RhoA transcription by reducing the c-Myc–Skp2–Miz1 transcriptional complex. These data document the fact that miR-34a suppresses RhoA activation at the initiation of transcription via targeting c-Myc. Our results demonstrate that miR-34a suppresses assembly and function of the c-Myc complex that activates transcription of RhoA.

The positive transcription elongation factor b (P-TEFb) regulates the promoter-proximal pause release of the elongation phase of transcription by Pol II [23]. c-Myc interacts with CycT1, the regulatory component of P-TEFb, and controls the elongation phase of transcription of Pol II [25], [26], [27]. P-TEFb is globally required for the generation of mRNAs and gene expression [27], [47] and is recruited to genes by a variety of transcription factors [23], [48]. Our results demonstrate that miR-34a suppresses assembly and function of the c-Myc complex that elongates transcription. It is known that P-TEFb is involved in aberrant transcriptional elongation in mixed-lineage leukemia (MLL) [49], [50]. Various tumor cell lines overexpress P-TEFb and CycT1 overexpression promotes transformation and tumor growth [51]. Our study demonstrates here that miR-34a abrogates the c-Myc-P-TEFb complex by targeting c-Myc. Accumulating evidence has shown the importance of P-TEFb in malignancy, thus linking miR-34a to P-TEFb is a significant finding in cancer biology. Our study partly gives an account of the global and profound effects of miR-34a on the cellular processes demonstrated here and in previous reports [4], [5], [7], [8].

As far as we know, the effects of microRNAs on the assembly of protein complexes have never been reported. Here we report the effects of miR-34a on the assembly of c-Myc functional complexes, the c-Myc–Skp2–Miz1 and c-Myc-P-TEFb complexes. c-Myc activates a variety of genes as part of a protein complex, regulates transcriptional elongation and mainly functions through different complexes with other proteins. The effects of miR-34a on these c-Myc functional complexes provide direct evidence of how miR-34a functions to suppress c-Myc. The c-Myc complexes which were induced by miR-34a were heterogeneous since the ratios of the components were different from those in the control immunocomplexes (Figs. 4C and 6A). These results indicate that the complexes were altered by miR-34a, suggesting a new complex assembly. The alteration of the c-Myc complexes by miR-34a also suggests that the suppression of the assembly of the c-Myc complexes may involve unknown mechanisms in addition to the simple reduction in quantity of c-Myc protein by miR-34a since miR-34a has dramatic effects on cellular processes.

In conclusion we have shown for the first time that miR-34a suppresses assembly and function of the c-Myc–Skp2–Miz1 complex that activates RhoA and the c-Myc-pTEFB complex that elongates transcription of various genes. Therefore, our study reveals a novel role of miR-34a in the regulation of transcription by c-Myc.

## Materials and Methods

### Ethics Statement

Formalin-fixed, paraffin-embedded (FFPE) prostate cancer samples were obtained from the San Francisco Veterans Affairs (VA) Medical Center. Written informed consent was obtained from all patients and the study was approved by the UCSF Committee on Human Research (Approval number: H9058-35751-01). All animal care was in accordance with the guidelines of the San Francisco Veterans Affairs Medical Center and the study was approved by the San Francisco VA IACUC (Protocol number: 08-003-01).

### Cell culture and transfection

Human prostate carcinoma cell lines, PC-3, LNCaP and DU145 and a non-malignant epithelial prostate cell line, RWPE-1, were purchased from The American Type Culture Collection (Manassas, VA). The prostate cancer cell lines were cultured in RPMI 1640 medium supplemented with 10% fetal bovine serum (FBS). RWPE-1 cell line was cultured in keratinocyte growth medium supplemented with 5 ng/mL human recombinant epidermal growth factor and 0.05 mg/mL bovine pituitary extract (Invitrogen, Carlsbad, CA)

Cells in 6-well plates were transfected with 30 nM pre-miR negative control (NC) or pre-miR-34a (Applied Biosystems, Foster City, CA) using Lipofectamine 2000 (Invitrogen), according to the manufacturer's instructions.

### Tissue samples

Formalin-fixed, paraffin-embedded (FFPE) prostate cancer samples were obtained from the San Francisco Veterans Affairs Medical Center. All slides were reviewed by a board certified pathologist for the identification of prostate cancer foci as well as adjacent normal glandular epithelium.

### Generation of stable miR-34a cell lines

An HIV-based lentiviral packaging system including a plasmid expressing miR-34a or vector control was purchased from GeneCopoeia (Rockville, MD). Lentivirus particles were produced by transfecting lentiviral expression plasmid into 293T cells. PC-3 cells were infected with the HIV-based lentivirus expressing miR-34a or vector control (1.5×10^6^ infectious units of virus (IFU) (in 20 µl), and the infected PC-3 cells were selected with puromycin (0.5 ug/ml).

### Soft agar colony formation assay

Soft agar colony formation assay was performed by seeding cells in a layer of 0.35% agar/RPMI/FBS over a layer of 0.5% agar/RPMI/FBS. RPMI/FBS was added every 5 days to continuously supply growth supplements to the cells. Cultures were maintained in a humidified 37°C incubator. On day 14 after seeding, colony forming efficiency was quantified by light microscopy.

### In vivo tumor growth

Suspensions of the stable miR-34a expressing cells or the control cells (1×10^7^ cells in 100 µl RPMI medium) were subcutaneously injected into female nude mice (strain BALB/c nu/nu; Charles River Laboratories, Inc., Wilmington, MA, 4–5 weeks old). Tumor volume was calculated on the basis of width (x) and length (y): x^2^y/2, where x<y.

### RNA extraction and quantitative real-time PCR

RNA was isolated using the RNeasy mini kit (Qiagen, Valencia, CA) according to the manufacturer's instructions. Reverse transcription reactions were performed using a Reverse Transcription System Kit (Promega, Madison, WI). Quantitative real-time PCR analysis was performed in triplicate with an Applied Biosystems Prism7500 Fast Sequence Detection System using TaqMan universal PCR master mix according to the manufacturer's protocol (Applied Biosystems). Levels of RNA expression were determined using the 7500 Fast System SDS software version 1.3.1 (Applied Biosystems).

### miRNA extraction and quantitative real-time PCR

Total RNA was extracted from laser capture microdissected (LCM) FFPE tissues and cultured cells using a miRNeasy FFPE kit (Qiagen, Valencia, CA) and an RNeasy mini kit (Qiagen) according to the manufacturer's instructions. Reverse transcription reactions were performed using a Reverse Transcription System Kit (Applied Biosystems Inc., Foster City, CA). Quantitative real-time PCR analysis was performed as described above.

### Plasmids

Putative target sites of the 3′UTR were cloned into the PmeI-XbaI site of the dual luciferase pmirGLO vector (Promega, Madison, WI). For mismatch constructs, 6 mismatches were introduced in the putative target site.

A human c-Myc expression vector was constructed by subcloning the full-length cDNA of c-Myc (Invitrogen, Carlsbad, CA) into the HindIII–XhoI site of the pCMV6-ENTRY vector (Origene).

c-Met expression vector (human ORF Myc-DDK-tagged ORF clone of Homo sapiens met proto-oncogene) was purchased from Origene (Rockville, MD).

### Cell viability assay

Cell viability was measured using CellTiter 96 Aqueous One Solution Cell Proliferation Assay (Promega, Madison, WI), a colorimetric assay which measures the activity of reductase enzymes. Cells were seeded at a density of 1×10^3^ cells per well in flat bottomed 96-well plates. At the indicated times, CellTiter 96 Aqueous One reagent was added to each well according to the manufacturer's instructions. Cell viability was determined by measuring the absorbance at 490 nm using a kinetic microplate reader (Spectra MAX 190; Molecular Devices Co., Sunnyvale, CA). Data are the mean ± standard deviation (SD) of 3 independent experiments.

### Apoptosis analysis

Apoptosis was measured using flow cytometry (Cell Lab Quanta SC, Beckman Coulter, Brea, CA) with Annexin-V-FITC/7-AAD labeling. Measurements were repeated independently three times.

### Transwell invasion assay

PC-3 cells were grown in DMEM containing 10% FBS. Culture inserts of 8-µm pore size (Transwell; Costar) were coated with Matrigel (BD Biosciences, San Jose) (100 µg per well) and placed into the wells of 24-well culture plates. In the lower chamber, 500 µl of DMEM containing 10% FBS was added and 1×10^5^ cells were seeded to the upper chamber. After 48 hours of incubation at 37°C with 5% CO2, the number of cells that had migrated through the pores was fixed with 10% formalin and stained with 0.05% Crystal Violet. Crystal Violet was solubilized with methanol and absorbance (540 nm) of the solution was measured by a kinetic microplate reader (Spectra MAX 190; Molecular Devices Co., Sunnyvale, CA). Data are the mean ± S.D. of 3 independent experiments.

### Western blot

Protein extracts were resolved by SDS-PAGE and transferred to polyvinylidene fluoride membranes (Hybond-P; GE Healthcare, Piscataway, NJ), followed by incubation with the indicated primary and secondary antibodies conjugated to horseradish peroxidise (GE Healthcare). Signals were detected using the ECL detection system (Amersham ECL plus Western Blotting detection system, Fairfield, CT). Antibodies against c-Myc, RhoA, Skp2 and GAPDH were purchased from Cell Signaling Technology (Danvers, MA). Antibodies against Miz1, Max, Cdk9, ARHGEF3 and ARHGEF18 were purchased from GeneTex (Irvine, CA). An antibody against CycT1 was purchased from Santa Cruz Biotechnology (Santa Cruz, CA).

### Luciferase reporter assay

Cells in 24-well plates were transfected with 30 nM pre-miR negative control (NC) or pre-miR-34a (Applied Biosystems) using Lipofectamine 2000 (Invitrogen), according to the manufacturer's instructions. Transfections of plasmids were perfomed using FuGENE HD (Roche Diagnosis, Basel, Switzerland) according to the manufacturer's instructions. All transfection experiments were performed in triplicate. Luciferase activity was assayed at 48 h after transfection, using a dual-luciferase reporter assay system (Promega).

### Chromatin immunoprecipitation (ChIP)

Chromatin immunoprecipitations were performed using the Chromatin Immunoprecipitation Assay Kit (Epigentek, Brooklyn, NY) according to manufacturer's instructions. DNA was sheared by sonication. A 1% portion of the sheared DNA–protein complex was used for an input DNA sample. Antibodies against c-Myc or RNA polymerase II (Millipore, Billerica, MA) or normal rabbit IgG (Millipore) was used for immunoprecipitation. Real-time PCR quantitation of ChIP was performed in triplicate, normalized by input, and expressed as a fold increase over the control. Primers used for real-time PCR were as follows: RhoA E-box5/6, 5′ -CTTCGCGTGCGTGAAGAGTTG-3′ and 5′-CATCCACTATTGCTCAGGAGC-3′; GAPDH promoter, 5′-TACTAGCGGTTTTACGGGCG-3′ and 5′-TCGAACAGGAGGAGCAGAGAGCGA-3′.

### Immunoprecipitation (IP)

Cells were lysed in buffer containing 250 mM NaCl, 50 mM HEPES pH 7.5, 0.1% Nonidet P40, 5 mM EDT and a protease inhibitor cocktail (Sigma-Aldrich, St. Louis, MO). Protein A/G-Sepharose beads (Santa Cruz Biotechnology) were added to the lysate for 60 minutes at 4°C for preclearance. The precleared lysate was incubated with an anti-c-Myc antibody (Cell Signaling Technology) or normal rabbit IgG (Millipore) and protein A/G-Sepharose beads overnight at 4°C. The protein A/G-Sepharose beads were washed with the lysis buffer. The proteins were separated by SDS-PAGE and analyzed by Western blot.

### Statistical analysis

Data are shown as mean values ± standard deviation (SD). The Student's t-test was used to compare the two different groups. P values of less than 0.05 were regarded as statistically significant (n = 3).

## Supporting Information

Figure S1 **miR-34a expression in PC-3 cells.** (A) PC-3 cells were transfected with 30 nM pre-miR negative control (NC) or pre-miR-34a. miR-34a expression at 72 h of the transfection was analyzed by real-time PCR and was normalized to that of the control (NC). *, P<0.05 compared with control. (B) PC-3 cells were infected with the HIV-based lentivirus expressing miR-34a or vector control, and the infected PC-3 cells were selected with puromycin. miR-34a expression was analyzed by real-time PCR and was normalized to that of the control. *, P<0.05 compared with control.
(TIF)

Figure S2 **miR-34a suppresses proliferation of prostate cancer cells.** (A) Prostate cancer cells were seeded at a density of 1.5×10^3^ cells per well in 96-well plates. The cells were transiently transfected with pre-miR negative control (NC) or pre-miR-34a and cell viability was assayed at the indicated times. *, P<0.05 compared with control. (B) PC-3 cells were transiently transfected with pre-miR negative control (NC) or pre-miR-34a for 72 h. miR-34a induces morphological changes in PC-3 cells.
(TIF)

Figure S3 **miR-34a induces apoptosis in PC-3 cells.** PC-3 cells were transfected with pre-miR negative control (NC) or pre-miR-34a for 3 days. PC-3 cells were stained with AnnexinV-FITC/7-AAD and apoptosis was analyzed by flow cytometry.
(TIF)

Figure S4 **miR-34a target sequences of c-Met and c-Myc.**
(TIF)

Table S1 **Clinical data of laser capture microdissected (LCM) prostate cancer tissues.**
(XLSX)
