# Supplementary figures and images for: MicroRNA-34a Modulates c-Myc Transcriptional Complexes to Suppress Malignancy in Human Prostate Cancer Cells
Source: PLoS One. 2012 Jan 3;7(1):e29722. doi: 10.1371/journal.pone.0029722 (PMC3250472; doi:10.1371/journal.pone.0029722)

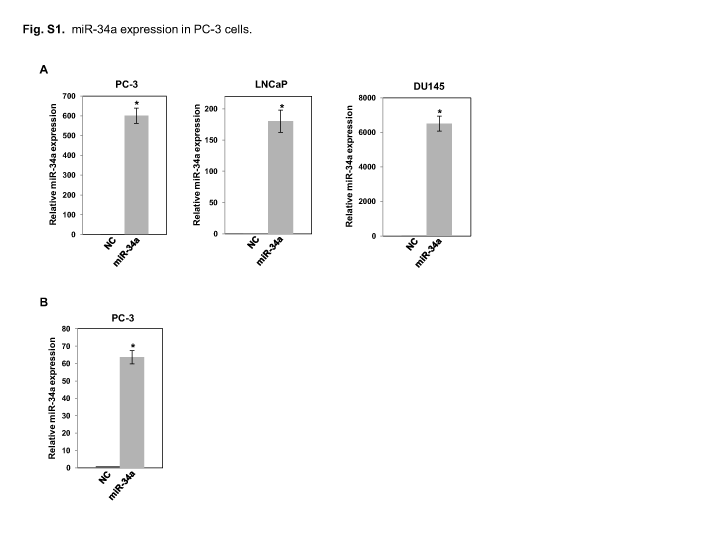

Supplement: Figure S1 — miR-34a expression in PC-3 cells. (A) PC-3 cells were transfected with 30 nM pre-miR negative control (NC) or pre-miR-34a. miR-34a expression at 72 h of the transfection was analyzed by real-time PCR and was normalized to that of the control (NC). *, P<0.05 compared with control. (B) PC-3 cells were infected with the HIV-based lentivirus expressing miR-34a or vector control, and the infected PC-3 cells were selected with puromycin. miR-34a expression was analyzed by real-time PCR and was normalized to that of the control. *, P<0.05 compared with control. (TIF) [file pone.0029722.s001.tif]

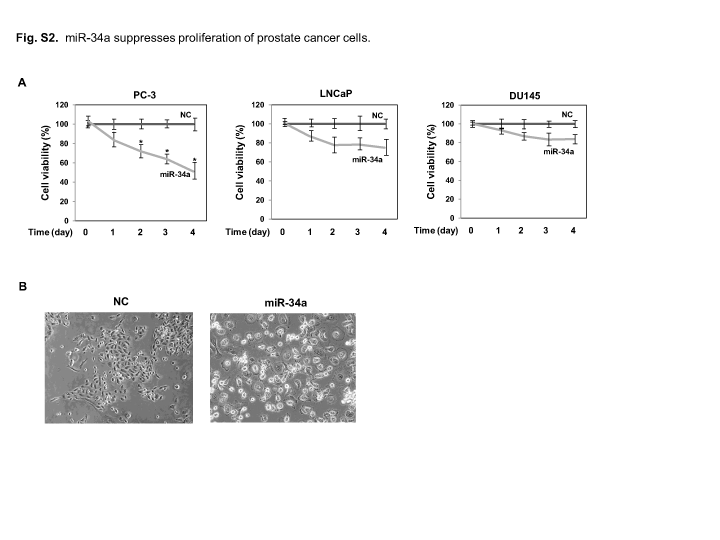

Supplement: Figure S2 — miR-34a suppresses proliferation of prostate cancer cells. (A) Prostate cancer cells were seeded at a density of 1.5×10^3^ cells per well in 96-well plates. The cells were transiently transfected with pre-miR negative control (NC) or pre-miR-34a and cell viability was assayed at the indicated times. *, P<0.05 compared with control. (B) PC-3 cells were transiently transfected with pre-miR negative control (NC) or pre-miR-34a for 72 h. miR-34a induces morphological changes in PC-3 cells. (TIF) [file pone.0029722.s002.tif]

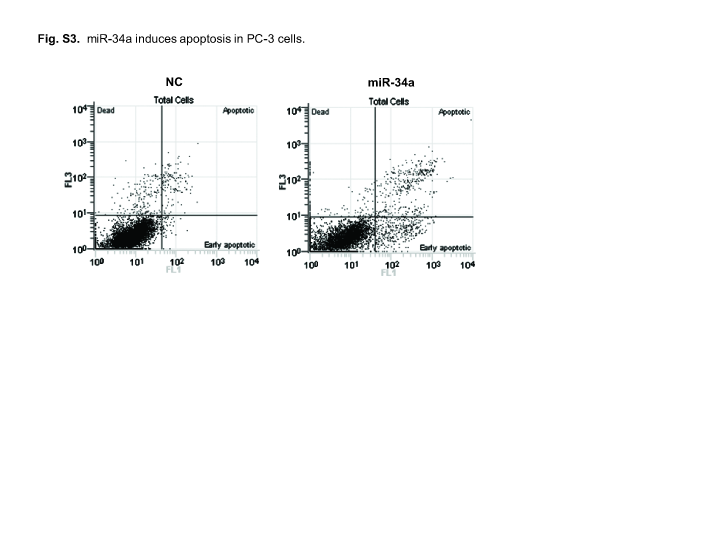

Supplement: Figure S3 — miR-34a induces apoptosis in PC-3 cells. PC-3 cells were transfected with pre-miR negative control (NC) or pre-miR-34a for 3 days. PC-3 cells were stained with AnnexinV-FITC/7-AAD and apoptosis was analyzed by flow cytometry. (TIF) [file pone.0029722.s003.tif]

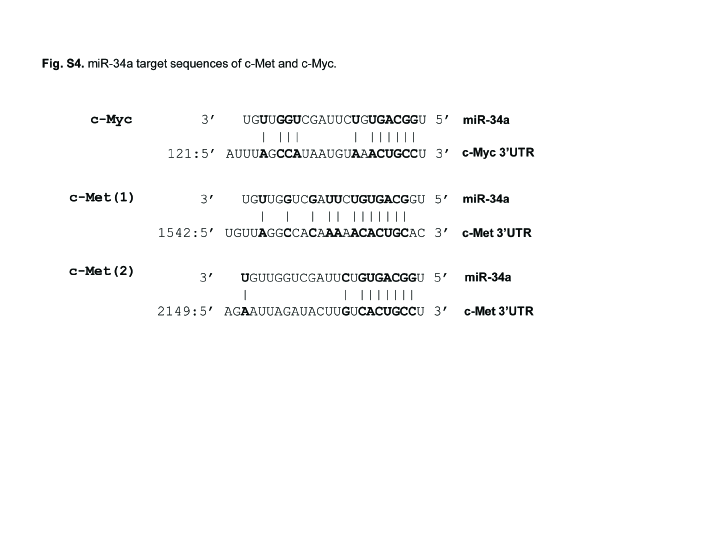

Supplement: Figure S4 — miR-34a target sequences of c-Met and c-Myc. (TIF) [file pone.0029722.s004.tif]
